# Selection and validation of optimal reference genes for RT-qPCR analyses in *Aphidoletes aphidimyza* Rondani (Diptera: Cecidomyiidae)

**DOI:** 10.3389/fphys.2023.1277942

**Published:** 2023-10-25

**Authors:** Xiu-Xian Shen, Guo-Qiang Zhang, Yu-Xin Zhao, Xiao-Xiao Zhu, Xiao-Fei Yu, Mao-Fa Yang, Feng Zhang

**Affiliations:** ^1^ Guizhou Provincial Key Laboratory for Agricultural Pest Management of the Mountainous Region, Institute of Entomology, College of Agriculture, Guizhou University, Guiyang, China; ^2^ Department of Entomology, College of Plant Protection, Nanjing Agricultural University, Nanjing, China; ^3^ College of Tobacco Science, Guizhou University, Guiyang, China

**Keywords:** *Aphidoletes aphidimyza*, chemosensory protein, expression stability, housekeeping genes, real-time quantitative PCR

## Abstract

*Aphidoletes aphidimyza* is a predator that is an important biological agent used to control agricultural and forestry aphids. Although many studies have investigated its biological and ecological characteristics, few molecular studies have been reported. The current study was performed to identify suitable reference genes to facilitate future gene expression and function analyses via quantitative reverse transcription PCR. Eight reference genes *glyceraldehyde-3-phosphate dehydrogenase* (*GAPDH*), *RPS13*, *RPL8*, *RPS3*, *α-Tub*, *β-actin*, *RPL32*, and *elongation factor 1 alpha* (*EF1-α*) were selected. Their expression levels were determined under four different experimental conditions (developmental stages, adult tissues, sugar treatment, and starvation treatment) using qRT-PCR technology. The stability was evaluated with five methods (Ct value, geNorm, NormFinder, BestKeeper, and RefFinder). The results showed that *GAPDH*, *RPL32*, and *EF1-α* were ranked as the best reference gene combinations for measuring gene expression levels among different developing stages and in various starvation treatments. *RPL8* and *RPS3* were recommended to normalize the gene expression levels among different adult tissues. *RPL32*, *β-actin*, and *EF1-α* were recommended sugar-feeding conditions. To validate the utility of the selected reference pair, *RPL8,* and *RPS3*, we estimated the tissue-biased expression level of a chemosensory protein gene (*AaphCSP1*). As expected, *AaphCSP1* is highly expressed in the antennae and lowly expressed in the abdomen. These findings will lay the foundation for future research on the molecular physiology and biochemistry of *A. aphidimyza*.

## Introduction

The *Aphidoletes aphidimyza* Rondani (Diptera: Cecidomyiidae) is widely utilized as an efficient predator of aphids in various agricultural systems, and its larvae can prey voraciously on more than 80 aphid species (Boulanger et al., 2019). To date studies on *A*. *aphidimyza* have focused on biological characteristics (Choi et al., 2004; Guo et al., 2014; Watanabe et al., 2016; De Azevedo et al., 2018; Madahi et al., 2019; Fratoni et al., 2020; Higashida et al., 2022), biological control (Lin et al., 2017; Shang et al., 2019), and the mitochondrial genome (Shen et al., 2022), but little is known about molecular mechanisms. Gene expression-level analysis is fundamental in the study of regulatory mechanisms of genes related to host selection, predation, drug resistance, stagnation, and neuromodulation. However, to date, no study investigating the aphid-eating *A*. *aphidimyza* gene expression profile has been published. To support further research on this economically valuable biological control species, basic research such as screening for *A*. *aphidimyza* reference genes is necessary.

Quantitative reverse transcription PCR (qRT-PCR) has become one of the most popular methods for studying quantitative gene expression because of its high sensitivity, specificity, and reproducibility (Derveaux et al., 2010; Bustin, 2000; Bustin et al., 2009). The accuracy of qRT-PCR analyses is influenced by many biological and technical factors (Bustin et al., 2009). Stable reference genes should be selected for a wide range of conditions, tissues or organs, and developmental stages (Xie et al., 2021; Xu et al., 2021; Yan et al., 2021). Ribosomal protein genes are often used as reference genes to normalize qRT-PCR data, as are *glyceraldehyde-3-phosphate dehydrogenase* (*GAPDH*), *β-actin*, and *elongation factor 1 alpha* (*EF1-α*) (Van Hiel et al., 2009; Ponton et al., 2011; Silva et al., 2012; Fu et al., 2013; Liu et al., 2016). However, organisms do not possess a universal reference gene (Vandesompele et al., 2002). Each candidate reference gene must be validated under specific experimental conditions in order to facilitate the acquisition of accurate results (Pfaffl et al., 2004).

In the current study *A. aphidimyza* reference genes were evaluated and selected based on stability under a wide range of conditions. Eight potential reference genes of *glyceraldehyde-3-phosphate dehydrogenase* (*GAPDH*), *RPS13*, *RPL8*, *RPS3*, *α-Tub*, *β-actin*, *RPL32*, and *elongation factor 1 alpha* (*EF1-α*) were assessed for expression stability by ΔCt, geNorm, NormFinder, and BestKeeper during different developmental stages, in different adult tissues, under sugar treatment conditions, and under starvation treatment conditions. Based on rankings produced by the aforementioned four statistical algorithms, an overall ranking for each experimental condition was then generated using RefFinder. The results will function as a foundation for subsequent studies investigating *A. aphidimyza* gene expression and gene function.

## Materials and methods

### Insect rearing

The *A. aphidimyza* strain used in this study was collected at the tobacco station in Leshan Town, Zunyi City, Guizhou Province, China, in May 2017. They were raised in an artificial climate chamber at 24°C ± 1°C, a 16:8 light-dark cycle, and 70% relative humidity. Megara japonica Matsumura on broad bean plants was utilized to feed the larvae, while honey was fed to adults.

### Experimental conditions and sample collection

#### Developmental stage

*A. aphidimyza* individuals at different development stages, including 10–20 first–third larvae, 10 pupae, and 8 adults (4 females and 4 males) were collected representing different developmental stages. Each experimental treatment included three biological duplicates. All experiments were performed with three independent biological replicates. Individuals of each sample were collected and kept in 1.5 mL centrifuge tubes and then were rapidly frozen in liquid nitrogen, and stored at −80°C before the total RNA extraction.

#### Adult tissues

A.*aphidimyza* adults were stunned by keeping them in a −20°C refrigerator for 3 min. Multiple tissue samples, including antennae (500 adults each), head (without antennae, 300 adults), thorax (without wings and legs, 200 adults), abdomen (100 adults), and legs (300 adults) were dissected on ice using a sterilized scalpel and forceps.

#### Sugar treatment

*A. aphidimyza* adults emerging 12 h after eclosion were divided into four feeding treatments: no other food was provided, aphid honeydew, 10% sugar solution, and purified water. Each treatment was conducted for 24 h. A total of 15 adults consisting of males and females were collected.

#### Starvation treatment

The third larvae of *A. aphidimyza* were collected and starved in 7.5-cm Petri dishes for 0, 1, 3, and 5 days (*n* = 10 per group).

### Total RNA extraction and cDNA synthesis

Total RNA was extracted from each sample using Trizol Reagent (Invitrogen, USA) in accordance with the manufacturer’s instructions. NanoDrop 2000 (NanoDrop Technology, United States) was used to assess the concentration and and purity of RNA in each sample, and the RNA samples with absorbance ratios of A260/A280 around 2.0 were selected for further analysis. RNA integrity was verified via 1.2% agarose gel electrophoresis. Lastly, the extracted RNA was digested by DNase I (Takara, Japan) to remove genomic DNA contamination. 1 μg of total RNA was used to synthesize cDNA using the Prime Script TMRT Reagent kit (Takara, Japan). The cDNA from each sample was stored at −20°C prior to the use of both PCR and RT-qPCR.

### Selection and identification of candidate reference genes

Eight candidate genes, namely, *GAPDH*, *RPL32*, *RPS13*, *β-actin*, *RPS3*, *EF1-α*, α -*Tub,* and *RPL8* were selected from the literature. The primers of *GAPDH*, *RPL32*, *RPS13*, *β*-*actin*, *RPS3*, *EF1-α*, α -*Tub,* and *RPL8* were designed based on the transcriptome data of *A. aphidimyza* (unpublished) with NCBI Primer-BLAST to design the primers (Ye et al., 2012) and primer sequences are designed in Supplementary Table S1. Candidate genes were amplified using the 2 × Phanta Max Master Mixes (Vazyme, China). The PCR amplification conditions were set as 95°C for 3 min, followed by 35 cycles of 95°C for 15 s, 57°C for 15 s, and 72°C for 30 s; final extension at 72°C for 5 min. All amplification products were purified from 1.5% agarose gels using a gel extraction kit (Watson Biotechnologies, Shanghai). The FastPure^®^ Gel DNA Extraction Mini Kit (Vazyme, China) was used to purify. The FastPure^®^ Gel DNA Extraction Mini Kit (Vazyme, China) was used to purify and were amplified using the 2 × Phanta Max Master Mixes. The pEASY^®^-Blunt Zero Cloning Kit (TransGen Biotech, Beijing, China) was used to subclone purified DNA. Subcloning products were sequenced in both directions (Tsing Ke Biotechnology Nanjing, China). Sequences resulting from these experiments were submitted to GenBank (Supplementary Table S1).

### Quantitative real-time PCR

Primer sequences were designed using NCBI Primer-BLAST in Supplementary Table S2. qRT-PCR reactions were conducted in accordance with the manufacturer’s instructions using TB Green^®^ Premix Ex Taq™ Tli RNaseH Plus (Takara, Japan) and the QuantStudio™ 7Pro Real-Time PCR System (Applied Biosystems, Life Technologies, Carlsbad, CA, United States). The reaction mixture consisted of a 20 μL mixture containing 10 µL of 2 × SYBR Green qPCR Master Mix, 0.4 µL of forwarding primer (10 Mmol L^−1^), 0.4 µL of reverse primer (10 Mmol L^−1^), 0.4 µL of ROX Reference Dye II (50X), 1 µL of cDNA template, and 7.8 µL of double-distilled water. The qRT-PCR reaction conditions were as follows: initial denaturation at 95°C for 30 s, followed by 40 cycles at 95°C for 3 s, and ended with an annealing step at 60°C for 30 s. A melting curve analysis was conducted in the 60°C–95°C temperature range to ensure the specificity of the primers. Three independent biological replicates were set. A standard curve was generated from the five-fold dilution series of cDNA, the slopes were analyzed, and the corresponding amplification efficiencies were calculated. RT-qPCR efficiency (E) was determined via the following equation:

$$E=\left(10^{\left[-1/\text{slope}\right]}-1\right)\times 100\%$$

### Expression stability of candidate reference genes under different treatments

Evaluations of the stability of selected reference genes were conducted using geNorm (Andersen et al., 2004), BestKeeper (Silver et al., 2006), NormFinder (Szabo et al., 2004), and the ΔCt method (Yang et al., 2015). Lastly, a comprehensive ranking of under different conditions was performed using the web-based tool “RefFinder” (Livak and Schmittgen, 2001; Shi and Zhang, 2016). P The optimal number of reference genes for accurate normalization of the target gene was determined by the variation value (Vn/Vn+1) calculated by geNorm. Pairwise variation (Vn/_n+1_ = 0.15) Vn/n + 1 < 0.15 indicates that the optimal number of reference genes is n, and Vn/_n+1_ > 0.15 indicates that the optimal number is n + 1 (Vandesompele et al., 2002).

### Stability verification of candidate reference genes

The chemosensory protein (CSP) of *A*. *aphidimyza* was selected as the target gene to verify the stability of candidate reference genes (GenBank: OP321094). The primer sequence of the target gene was as follows:

Forward: AAC​GCT​TTT​GTT​GGA​CAG​CTA​C.

Reverse: CAA​TGA​ATC​GAA​GCA​CAC​GA.

Based on the stability (*RPL8* and *RPS13*) and instability (*β-actin*) of primary reference genes The average relative expressions of *AaphCSP1* in different female tissues were computed based on the 2^−ΔΔCT^ method (Livak and Schmittgen, 2001) and three independent biological replicates.

## Results

### Selection of candidate reference gene

Eight candidate genes of *A. aphidimyza* with a complete open reading frame (ORF) were identified by RT-PCR. The *GAPDH*, *RPL32*, *RPS13*, *β-actin*, *RPS3*, *EF1-α*, α -*Tub,* and *RPL8* genes had a 936, 387, 375, 1,081, 681, 1,260, 1,194, and 669 base pair (bp), separately. The obtained sequences were submitted to the GenBank database and the accession numbers are shown in Supplementary Table S1. The products from qRT-PCR were confirmed by sequencing. Melting curve analysis using the RT-qPCR of the eight candidate reference genes had a single peak, indicating the good specificity of the primers. The PCR efficiency (E) and the regression coefficient (*R* ^2^) were calculated using the slope of the standard curve established for each primer pair. The E-values ranged from 95.90% ( α -*Tub*) to 108.79% (*GAPDH*), which was within the required range of 80.0%–120.0% (Supplementary Table S2). The regression coefficient ranged from 0.994 (*EF1-α*) to 1.000 (*RPS13*) (Supplementary Table S2).

### Expression variations of candidate reference gene

**FIGURE 1:**
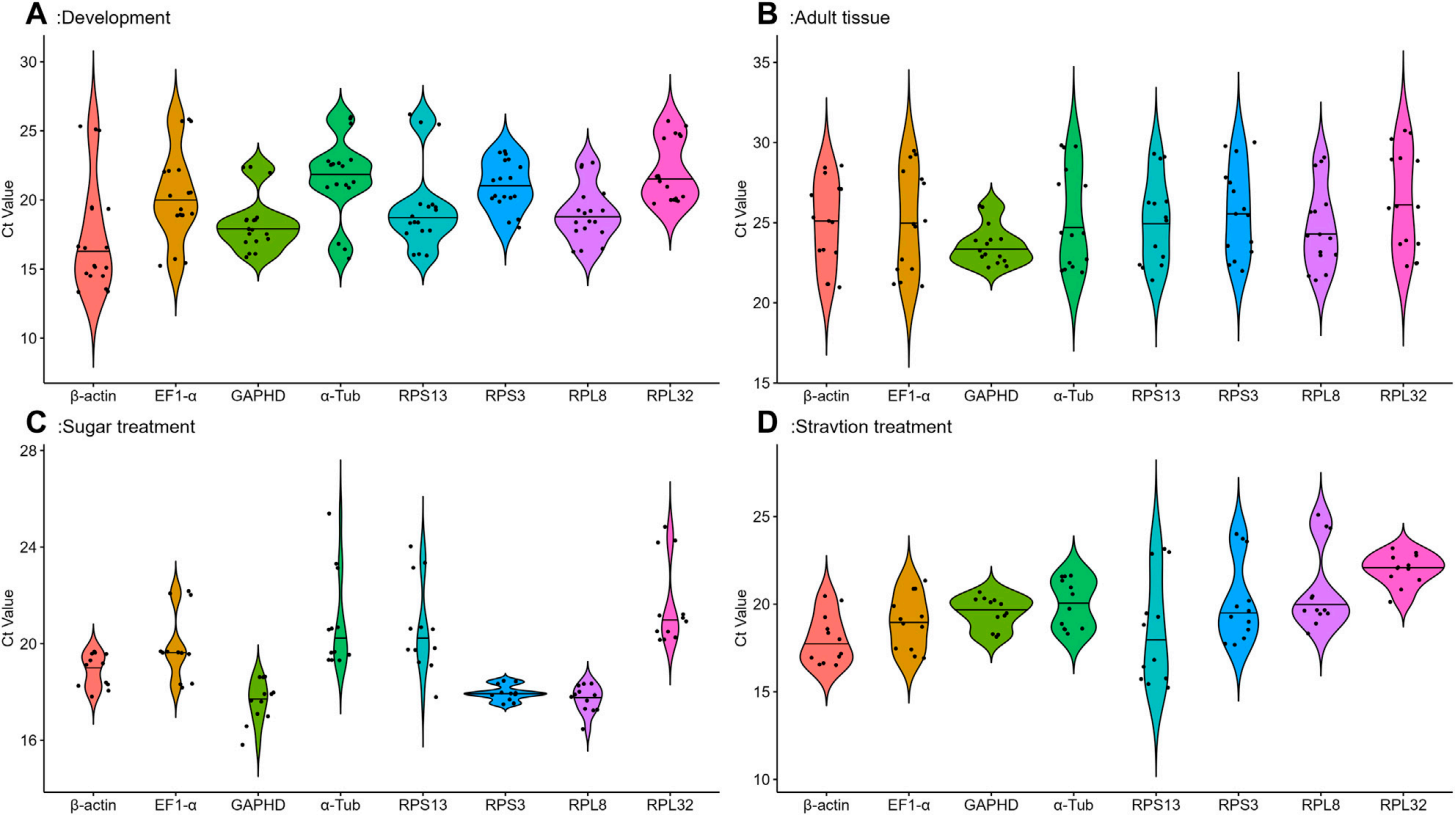
Expression levels of eight housekeeping genes in *A. aphidimyza* were investigated in four different experiments; **(A)** (developmental stages), **(B)** (adult tissues), **(C)** (sugar treatments), and **(D)** (starvation treatments). Mean Ct values for the eight candidate reference genes are presented in box plots, where each box indicates the 25th and 75th percentiles, and the line across the box represents the median. The eight genes analyzed were *GAPDH*, *RPL32*, *RPS13*, *β-actin*, *RPS3*, *EF1-α*, *α-Tub*, and *RPL8*.

Eight candidate reference genes were analyzed by qRT-PCR using cycle threshold values (Ct) reflecting their expression under different conditions.The gene expression analysis of the eight candidate reference genes in all samples under four conditions showed a range of Ct means of 13.34 (*β-actin*) −30.75 (*RPL32*) (Figure 1). At different developmental stages, *GAPDH* and *RPPL8* had the smaller gene expression variation, whereas *β-actin* and *EF1-α* had the higher expression difference (Figure 1A). Among various tissues, except for *GAPDH*, the expression fluctuations were higher in selected reference genes (Figure 1B). Adults feeding on different sugar varieties, *RPPL8* and *RPS3* had smaller gene expression variation (Figure 1C). Larvae starvation treatment conditions, *GAPDH* had a smaller gene variation (Figure 1D).

### Expression stability of candidate reference gene

#### Developmental stages

**FIGURE 2:**
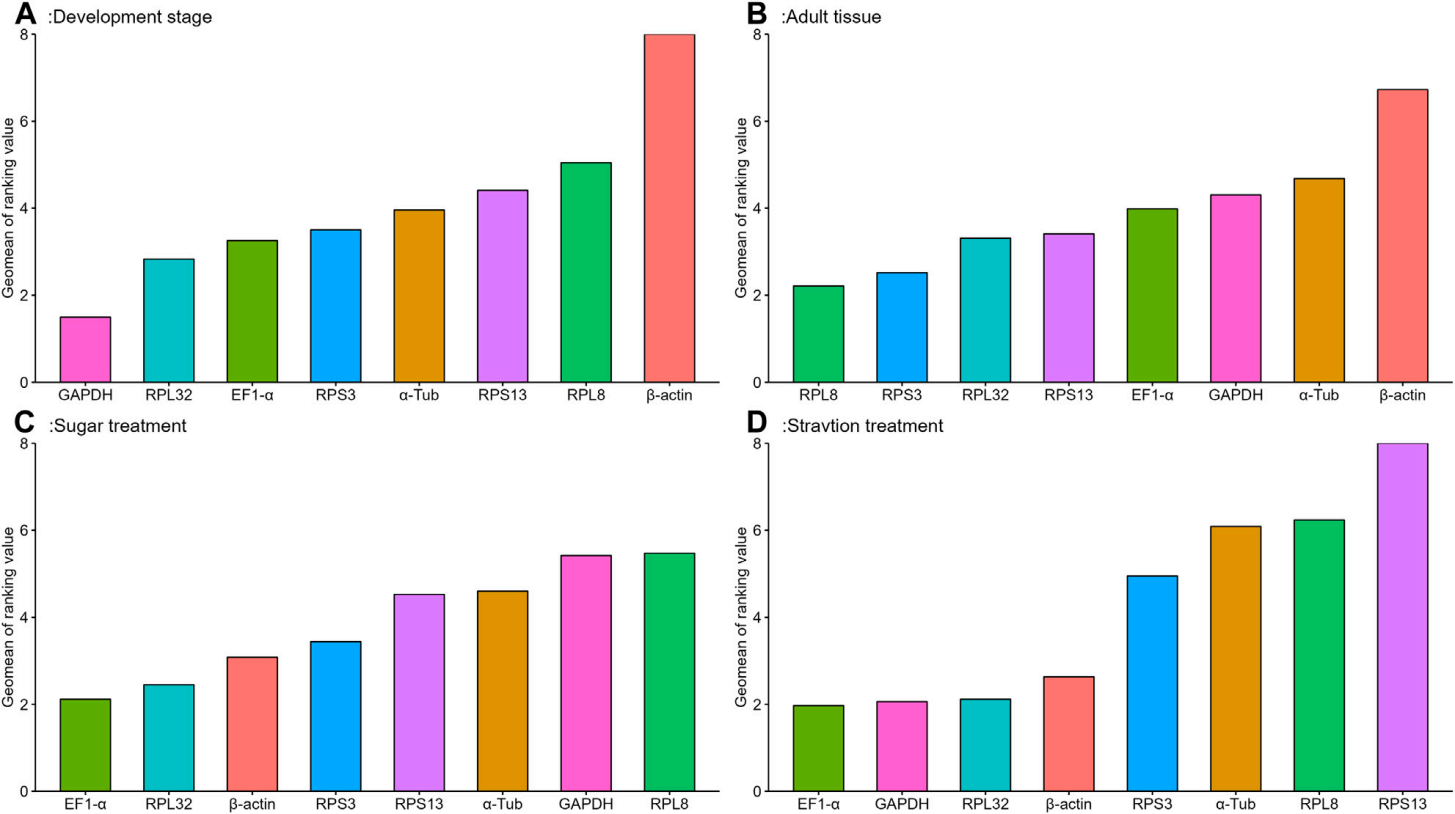
Stability of expression analysis of eight candidate reference genes of *A.aphidimyza* in four different types of experiments calculated by RefFinder.

The geNorm algorithm evaluates the candidate reference genes based on their expression stability values (M-values) and pairwise variations (Vn/Vn+1). The expression stability values revealed that *α-Tub* and *EF1-α* were the better reference genes during different developmental stages, with M-values below 1 (Table 1; Supplementary Figure S1). Pair-wise variation analysis of reference genes showed that V2/3 was less than 0.15 (Supplementary Figure S2), indicating that gene expression analysis required two different reference genes in the developmental stage. Based on the above comprehensive ranking, we recommended the following two genes as reference genes in developmental stages: *GAPDH* and *RPS13* (Supplementary Figure S2). According to the NormFinder, the stable gene was *GAPDH*, with a *p*-value less than 0.2. The most unstable gene was *β-actin*, with a *p*-value of 0.314 (Table 1; Supplementary Figure S1). Based on the BestKeeper analysis, *GAPDH* was the most stable gene (Table 1; Supplementary Figure S1). The stability of the eight *A. aphidimyza* candidate reference genes were ranked by RefFinder at various developmental stages from high to low: *GAPDH* > *RPL32* > *EF1-α* > *RPS3* > *α-Tub* > *RPS13* > *RPL8* > *β-actin* (Figure 2A). Therefore, *GAPDH* and *RPS13* are ranked as the best reference gene combinations for measuring target genes among different developing stages (Figure 2).

**TABLE 1:** Stability of the expression of eight candidate reference genes under different experimental conditions.

Condition	Rank	geNorm		Normfinder		BestKeeper		ΔCt	
		Gene name	SV	Gene name	SV	Gene name	SD	Gene name	SV
Developmental stages	1	*EF1-* α	0.880	*GAPDH*	0.820	*GAPDH*	1.458	*GAPDH*	1.766
	2	α *-Tub*	0.880	*RPL32*	1.006	*RPS3*	1.508	*RPL32*	1.855
	3	*RPS3*	1.416	*RPS13*	1.429	*RPL8*	1.521	*RPS13*	2.017
	4	*RPL32*	1.575	*EF1-* α	1.465	*RPL32*	1.932	*EF1-* α	2.046
	5	*GAPDH*	1.681	RPS3	1.519	α *-Tub*	2.106	*RPS3*	2.059
	6	*RPL8*	1.842	*RPL8*	1.549	*RPS13*	2.167	*RPL8*	2.082
	7	*RPS13*	1.928	α *-Tub*	1.897	*EF1-* α	2.476	α *-Tub*	2.269
	8	β *-actin*	2.078	β *-actin*	2.184	β *-actin*	3.266	β *-actin*	2.527
Adult tissue	1	*RPL32*	0.266	*RPL8*	0.070	*GAPDH*	0.960	*RPS13*	1.025
	2	*EF1-* α	0.266	*RPS3*	0.413	*RPL8*	2.109	*RPL8*	1.049
	3	α *-Tub*	0.470	*RPS13*	0.564	*RPS3*	2.118	*RPS3*	1.106
	4	*RPS3*	0.579	α *-Tub*	0.674	β *-actin*	2.240	*RPL32*	1.112
	5	*RPS13*	0.677	*RPL32*	0.762	*RPS13*	2.310	α *-Tub*	1.147
	6	*RPL8*	0.711	*EF1-* α	0.901	*RPL32*	2.730	*EF1-* α	1.183
	7	*GAPDH*	1.055	GAPDH	1.672	*EF1-* α	2.759	*GAPDH*	4.097
	8	β *-actin*	1.342	β *-actin*	2.038	α *-Tub*	2.777	β *-actin*	2.201
Sugar treatment	1	α *-Tub*	0.464	*EF1-* α	0.433	*RPS13*	0.231	*EF1-* α	1.043
	2	*RPL32*	0.464	β *-actin*	0.771	*RPL8*	0.433	*RPL32*	1.179
	3	*RPS13*	0.521	*RPL32*	0.896	β *-actin*	0.620	β *-actin*	1.189
	4	*EF1-* α	0.602	*RPS3*	1.009	*GAPDH*	0.667	*GAPDH*	1.279
	5	β *-actin*	0.954	*RPS13*	1.025	*EF1-* α	1.091	*RPS13*	1.294
	6	*GAPDH*	1.137	GAPDH	1.038	*RPL32*	1.413	*RPS3*	1.310
	7	*RPS3*	1.222	RPL8	1.192	*RPS3*	1.431	*RPL8*	1.351
	8	*RPL8*	1.268	α *-Tub*	1.208	α *-Tub*	1.533	α *-Tub*	1.443
Starvation treatment	1	*GAPDH*	0.370	*EF1-* α	0.302	*RPL32*	0.672	*EF1-* α	1.416
	2	*RPL32*	0.370	β *-actin*	0.438	*GAPDH*	0.736	β *-actin*	1.469
	3	*EF1-* α	0.882	*GAPDH*	0.943	β *-actin*	1.169	*GAPDH*	1.512
	4	β *-actin*	1.054	*RPS3*	1.150	α *-Tub*	1.250	*RPL32*	1.616
	5	*RPS3*	1.294	RPL32	1.179	*EF1-* α	1.252	*RPS13*	1.710
	6	α *-Tub*	1.421	*RPL8*	1.463	*RPS13*	1.846	*RPL8*	1.883
	7	*RPL8*	1.567	α *-Tub*	1.989	*RPL8*	1.905	α *-Tub*	2.159
	8	*RPS13*	1.766	RPS13	2.150	*RPS3*	2.595	*RPS3*	2.362

#### Adult tissue

Based on the geNorm algorithm, the M-values of *EF1-α* and *RPL32* were below 0.4 (Table 1; Supplementary Figure S1). The pairwise variation analysis displayed that the V2/3 values were close to 0.15 (Supplementary Figure S2), indicating that two reference genes should be selected as reference genes in subsequent studies on other genes in adult tissues. We recommended *RPS13* and *RPL8* as reference genes (Supplementary Table S3). Based on the BestKeeper, *GAPDH* was the most stable gene. According to the NormFinder, *RPL8* was the most stable gene (Table 1; Supplementary Figure S1). Combining the four algorithms, the comprehensive ranking by RefFinder was as follows: *RPL8* > *RPS3* > *RPL32* > *RPS13* > *EF1-α > GAPDH* > *α-Tub* > *β-actin* (Figure 2B). Therefore, *RPL8* and *RPS3* are ranked as the best reference gene combinations for measuring target genes among different adult tissues (Figure 2).

#### Sugar treatment

In this study, the geNorm algorithm results showed that the comprehensive reference gene rankings from the best to the least stable were *α-Tub*, *RPL32*, *RPS13*, *EF1-α*, *β-actin*, *GAPDH*, *RPS3*, and *RPL8* (Table 1; Supplementary Figure S1). Except for *GAPDH*, *RPS3*, and *RPL8*, the other genes in the selected reference genes showed values below 1, indicating their stabilities were similar. Moreover, the pairwise variation analysis showed that the V2/3 value exceeded 0.15, indicating three different reference genes are needed for gene expression analysis in sugar treatment (Supplementary Figure S2). The NormFinder analysis revealed that the stability of the selected reference genes was *EF1-α*, *β-actin*, and *RPL32*, with *p*-values of 0.433, 0.771, and 0.896, respectively, indicating their similar stability (Table 1; Supplementary Figure S1). The BestKeeper data showed that *RPS13*, *RPL8*, *β-actin*, and *GAPDH* were the most stable because they showed Cp values of 0.231, 0.433, 0.620, and 0.667, respectively (Table 1; Supplementary Figure S1). The stability of reference genes was evaluated comprehensively using RefFinder, which ranked them as *EF1-α* > *RPL32* > *β-actin* > *RPS3* > *RPS13* > *α-Tub > GAPDH* > *RPL8* (Figure 2C). Thus, the three reference genes (*EF1-α*, *RPL32*, and *β-actin*) are recommended to be used to test the target gene expression levels in sugar treatment.

### Starvation treatment

Based on the geNorm algorithm, the M-values of *GAPDH*, *EF1-α*, and *RPL32* were below 0.4 (Table 1; Supplementary Figure S1). The pairwise variation analysis displayed that the V2/3 values exceeded 0.15, suggesting three reference genes are enough for gene expression determination within starvation treatment (Supplementary Figure S2). The NormFinder analysis revealed that the stability of the selected reference genes was *EF1-α* > *β-actin* > *GAPDH* > *RPS3* > *RPL32* > *RPL8 > α-Tub* > *RPS13*, with the *p*-value of 0.302, 0.438, 0.943, 1.150, 1.179, 1.463, 1.989, and 2.150, respectively. Again, the *p* values of *EF1-α* and *β-actin* were below 0.5 (Table 1; Supplementary Figure S1), indicating their similar stability. The BestKeeper data revealed that *RPL32* and *GAPDH* were the most stable because they showed Cp values of 0.672 and 0.736, respectively. The Cp values of *β-actin*, *α-Tub*, *EF1-α*, *RPS13,* and *RPL8* were more than 1.0, and the Cp value of *RPS3* was more than 2.0 (Table 1; Supplementary Figure S1). The RefFinder showed a comprehensive ranking order from the most to the least stable: *EF1-α* > *GAPDH* > *RPL32* > *β-actin* > *RPS3* > *α-Tub > RPL8* > *RPS13* (Figure 2D). Thus, we considered *EF1-α*, *GAPDH*, and *RPL32* as the most appropriate reference gene combinations.

### Verification of candidate reference genes

**FIGURE 3:**
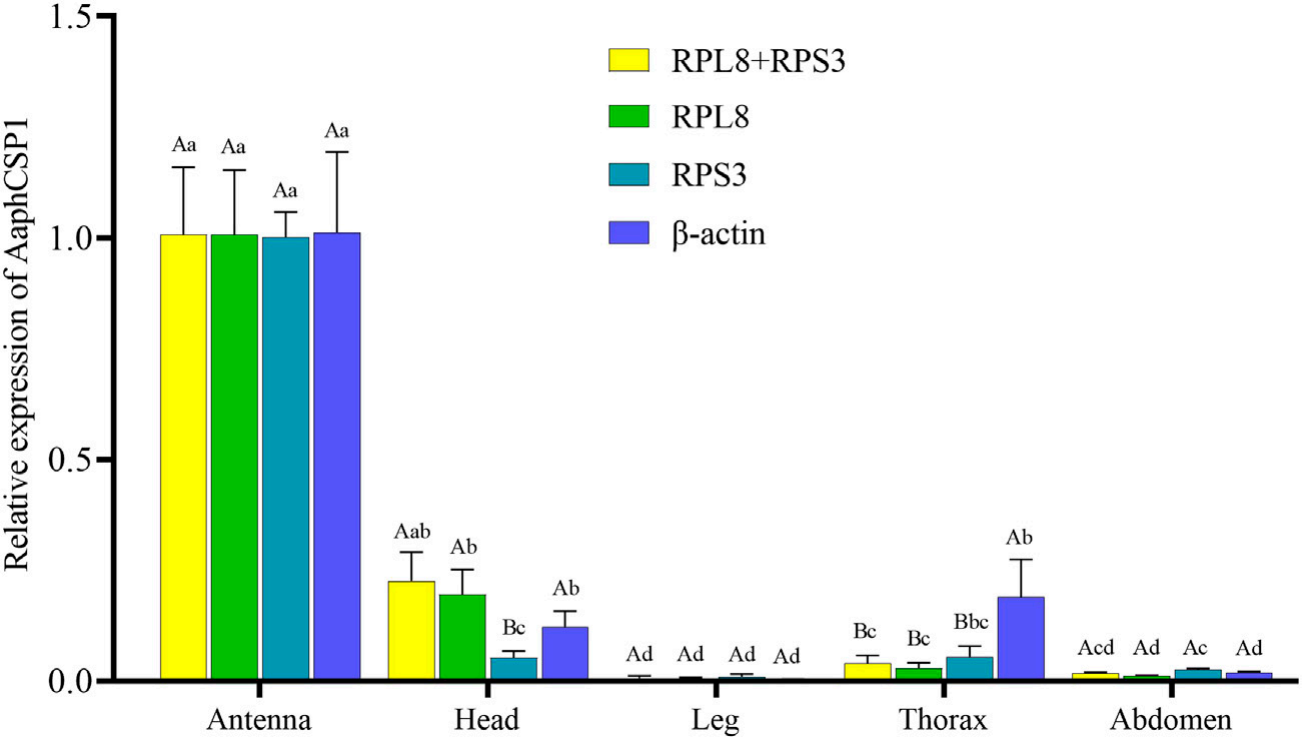
Expression of *AaphCSP1* gene under different female tissue. Three reference gene combinations (*RPL8* + *RPS3*, *RPS3*, *RPL8*, *β*-actin) were used for the normalization. The values were calculated using the 2^−ΔΔCT^ method. The relative transcripts are the ratios of copy numbers in different tissue relative to the antenna, which is set as 1. The data in the figure were the mean ± standard error. Different lowercase letters indicate that, after normalization with the same reference gene, there was a significant difference in the expression level of *AaphCSP1* in female adult *A. aphidimyza* (Tukey’s HSD-p < 0.05). Different uppercase letters indicate that there were significant differences in the normalization results of each reference gene (Tukey’s HSD-p < 0.05).

To evaluate the stability of the selected reference genes, we selected *AaphCSP1* as the target gene and analyzed the expression level of *AaphCSP1* in the antenna, head, leg, thorax, and abdomen. The following reference genes were used to normalize: *RPL8*, *RPS3*, *RPL8* + *RPS3* (the most stable reference gene), and *β-actin* (the least stable reference gene). The highest accumulated mRNA level of *AaphCSP1* was found in the antenna, followed by those in the head, the lowest level was detected in the leg, thorax, and abdomen. However, *β-actin* was used as a reference gene, and *AaphCSP1* was highly expressed in the thorax exceeded (*p* < 0.05) (Figure 3).

## Discussion

Quantitative real-time PCR is a crucial technique for studying gene expression analysis. This technique uses a quantitative method to detect gene expression, with the internal reference gene serving as a standard to calibrate the expression of the target gene. It is highly sensitive and has good repeatability, making it a standard technique for detecting or comparing mRNA levels in gene expression studies. The expression level of any single reference gene does not remain constant under all experimental treatments however, so appropriate reference genes must be chosen before RT-qPCR is performed (Shi and Zhang, 2016). Reference genes have been screened in a variety of insects, such as *Bombyx mori* (Peng et al., 2012), *Podoptera frugiperda* (Zhou et al., 2021), and *Anastatus japonicus* (Liu et al., 2022).

The stability evaluation of candidate reference genes is often performed by multiple methods based on a comprehensive analysis of relevant parameters, and the candidate reference genes are ranked according to their stability. In the present study five methods were used individually to rank stability at various developmental stages and in different tissues of *A. aphidimyza* adults, and the most stable reference genes and their rankings were not entirely consistent. For example, BestKeeper and ΔCt determined that *GAPDH* was the most stable gene during the various development stages of *A. aphidimyza* development, whereas GeNorm determined that *EF1-α* = *α-Tub* was the most stable gene, and NormFinder determined that *GAPDH* was the most stable gene (Table 1; Supplementary Figure S1). Similar discrepancies were observed among different adults tissues, sugar treatment conditions, and starvation treatment conditions. To lessen disparities in outcomes between software, RefFinder was used to synthetically rate the stability of each possible reference gene (Figure 2). Similar situations have been observed in the endogenous screening of other insects (Yang et al., 2017).

In different adult tissues, the reliable reference genes were *RPS3* and *RPL8* (Table 1; Figure 2). Ribosomal proteins are essential housekeeping components that regulate the synthesis of cellular ribosomes, and participate in cellular translation processes. Members of the ribosomal gene family have been used as reference genes in many studies. For example, *RPS18* had the highest expression stability across tissues in *Tetranychus cinnabarinus* (Sun et al., 2010), *RPS11* had the highest expression stability in *Nilaparvata lugens* (Yuan et al., 2014), and *RPS13* had the highest expression stability in *Sesamia inferens* (Sun et al., 2015). *RPS15* and *RPL13* were also reported as a stable pair of reference genes in different tissues of larvae of the cotton bollworm *Helicoverpa armigera* (Zhang et al., 2015). In *Mythimna separata* larvae *RPL12* was the most stable gene in different tissues (Li et al., 2018). *RPS13* had the highest expression stability in different adult tissues of *Tuta absoluta* (Yang et al., 2021), whereas *RPL13* exhibited higher stability in specific larval tissues of *Phthorimaea operculella* (Shen et al., 2022).

*GAPDH* remained stable during developmental stages and under sugar treatment conditions in adult *A*. *aphidimyza* (Table 1; Figure 2). The *GAPDH* enzyme regulates glycolysis, gluconeogenesis, and other mechanisms of energy metabolism. For example, *GAPDH* was the most stable reference gene during various developmental stages in *Schistocerca gregaria* (Van Hiel et al., 2009), *Spodoptera litura* (Lu et al., 2013), *Spodoptera exigua* (Zhu et al., 2014), and *Sesamia inferens* (Lu et al., 2015). However, numerous investigations in different insects have revealed that *GAPDH* expression can occasionally be erratic, such as in *Harmonia axyridis* (Qu et al., 2018), *Tetranychus cinnabarinus* (Sun et al., 2010), *Sogatella furcifera* (An et al., 2016), *Bombus terrestris*, and *Bombus lucorum* (Horňáková et al., 2010). *EF1-α* has been identified as a highly stable reference gene in various insect species. In both *Thitarodes armoricanus* larvae (Liu et al., 2016) and *Danaus plexippus* (Pan et al., 2015) *EF1-α* is evidently insensitive to changes in tissue type or developmental stage. *EF1-α* has exhibited good expression stability in *Phthorimaea operculella* during several developmental stages and at different temperatures (Shen et al., 2022). Notably, under numerous sugar treatment conditions in the current study *EF1-α* was the most reliable reference gene in adult *A. aphidimyza* (Table 1; Figure 2).

*β-actin* is a highly conserved cytoskeletal protein that plays a crucial role in various eukaryotic physiological processes such as cell division, chromosome movement, organelle movement, and cytoplasmic flow. It has been identified as one of the most stable reference genes in several insect species, including *Liriomyza trifolii* (Chang et al., 2017), and *Drosophila melanogaster* (Ponton et al., 2011). However, in the present study, it was the worst reference gene in different tissues and under different sugar treatment conditions in adult *A. aphidimyza* (Table 1; Figure 2). This observation is consistent with previous reports that *β-actin* was less stable across temperature and photoperiod treatments in *Helicoverpa armigera* (Shakeel et al., 2015), and in Coleopteran insect species such as *Coccinella septempunctata* (Yang et al., 2016), *Henosepilachna vigintioctomaculata* (Lü et al., 2018), *Phaedon brassicae* (Ma et al., 2021), and *Phthorimaea operculella* (Shen et al., 2022). *α-Tub* is a gene that encodes microtubule proteins involved in a number of physiological processes such as the construction and maintenance of cell morphology, intracellular transport, chromosome movement, and cell division. It is widely considered to be a reliable reference gene in several insect species, including *Chilo suppressalis* larvae (Xu et al., 2019) and different developmental stages of *Lymantria dispar* (Yin et al., 2020). In the present study however, in different tissues and under different starvation treatment conditions in *A. aphidimyza*, *α-Tub* was the worst reference gene (Table 1; Figure 2).

In order to demonstrate the utility of *RPL8* and *RPS3* inaccurate gene expression analysis in *A. aphidimyza*, we evaluated the relative gene expression level of *AaphCSP1* in the antenna, head, leg, thorax, and abdomen. Our results showed that *AaphCSP1* was abundantly expressed in the antenna, moderately transcribed in the head, and lowly expressed in the thorax and abdomen (Figure 3). Our expression data are consistent with the fact that CSPs are highly expressed in pest and parasite antennae, their main function is thought to be chemoreception (Liu et al., 2012). Including pheromone detection (Angeli et al., 1999), reproduction (Zeng et al., 2020), and host hunting (Peng et al., 2021). Thus, the tissue-biased expression pattern of *AaphCSP1* demonstrates that *RPL8* and *RPS3* can be used as endogenous controls to assess gene expression in *A. aphidimyza*.

## Data Availability

The datasets presented in this study can be found in online repositories. The names of the repository/repositories and accession number(s) can be found in the article/Supplementary Material.

## Supplementary material

SUPPLEMENTARY FIGURE S1 Stability of expression of eight candidate reference genes in *A. aphidimyza* calculated by three different software programs (geNorm, NormFinder, and BestKeeper). **(A)** Different developmental stages, **(B)** adult tissues, **(C)** sugar treatment conditions, and **(D)** starvation treatment conditions.

SUPPLEMENTARY FIGURE S2 Evaluation of the optimal number of reference genes for normalization of *A. aphidimyza* in different treatments, determined via geNorm. The dashed line indicates that the pairwise variation is 0.15. Vn/_n + 1_ < 0.15 indicates that the optimal number of reference genes for normalization is n, and the Vn/_n + 1_ > 0.15 indicates that the optimal number is n + 1.
