# Supplementary figures and images for: Selection and validation of optimal reference genes for RT-qPCR analyses in Aphidoletes aphidimyza Rondani (Diptera: Cecidomyiidae)
Source: Front Physiol. 2023 Oct 25;14:1277942. doi: 10.3389/fphys.2023.1277942 (PMC10634233; doi:10.3389/fphys.2023.1277942)

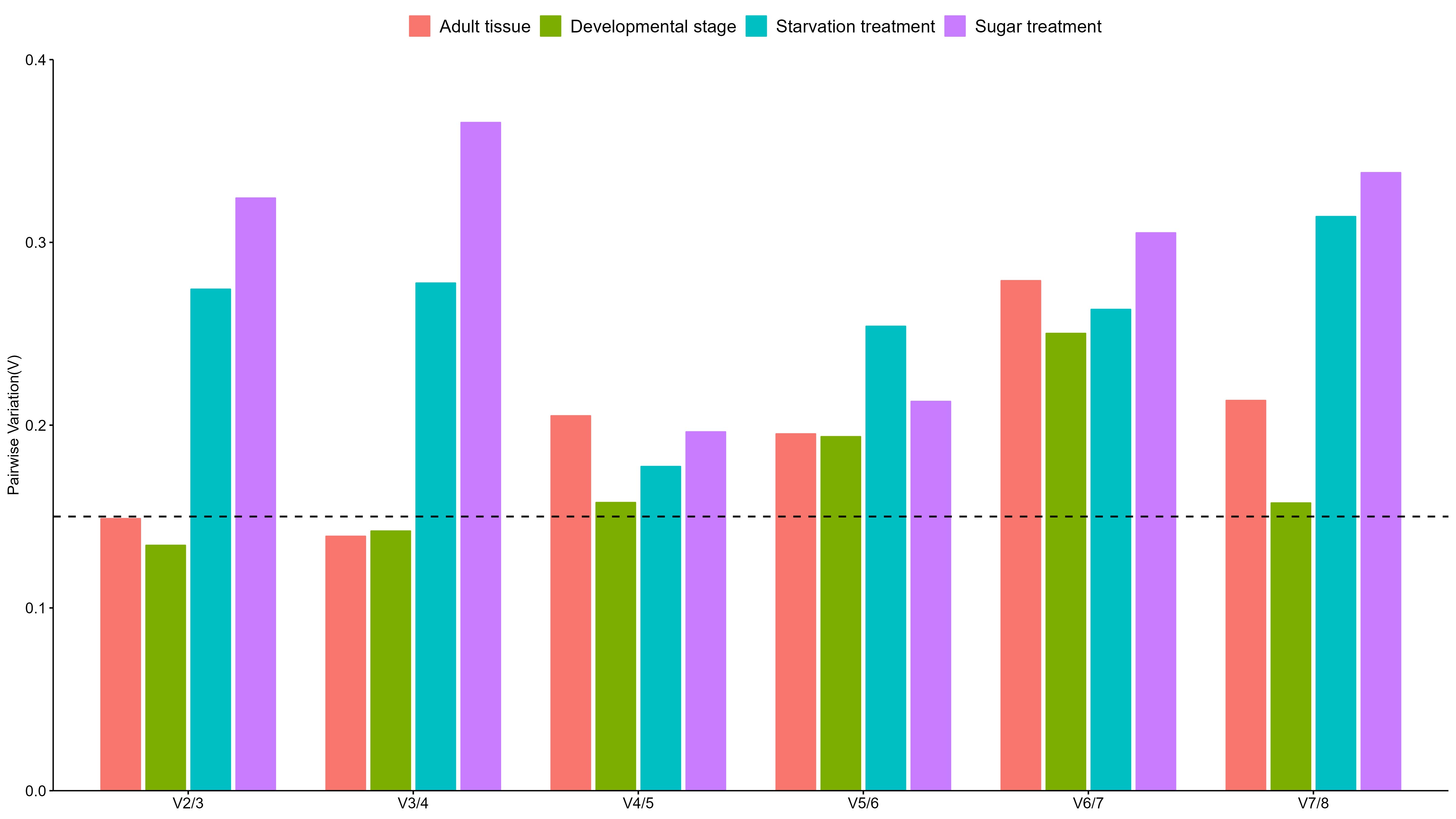

Supplement: Supplementary file 1 [file Image2.jpg]

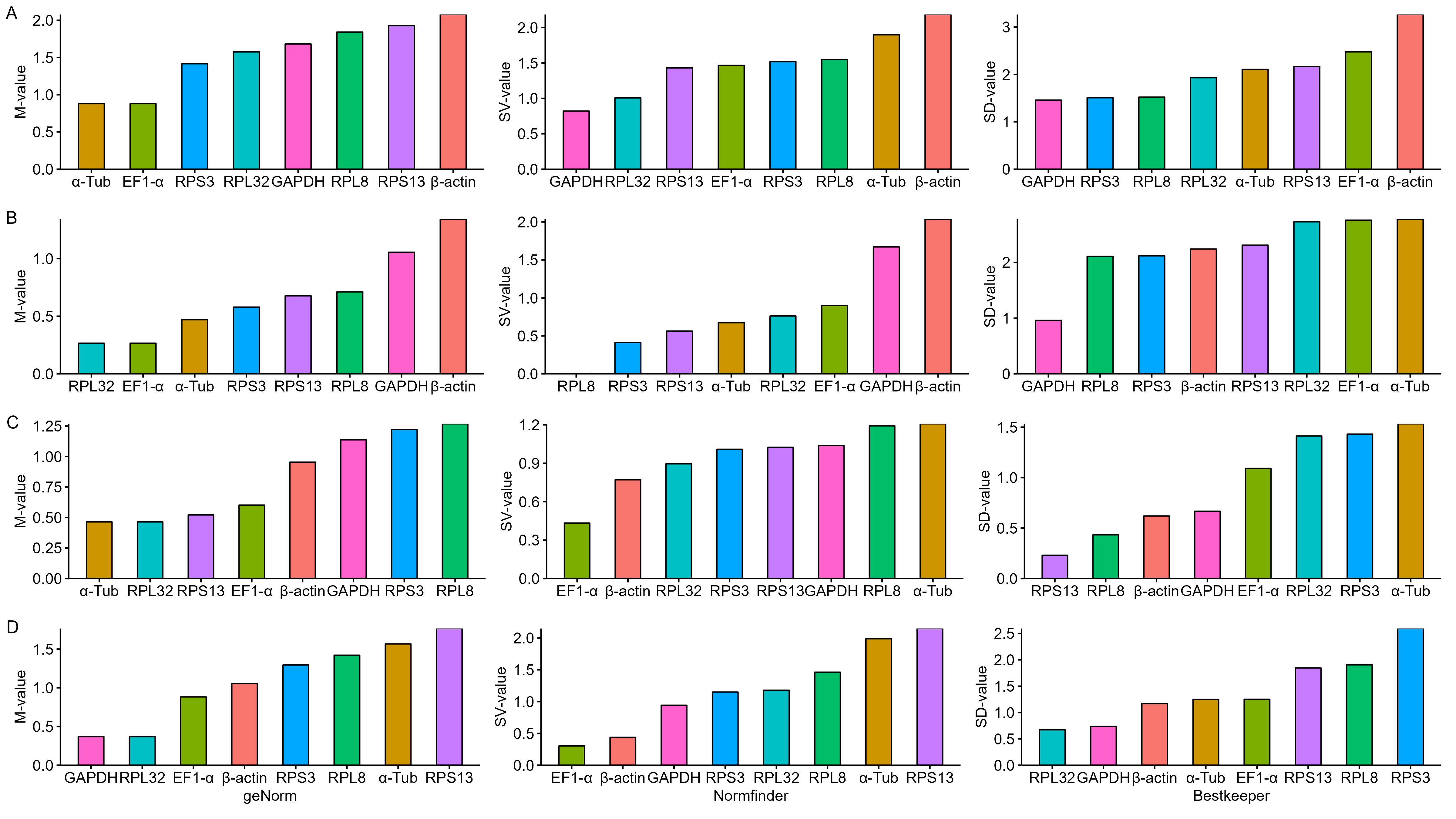

Supplement: Supplementary file 4 [file Image1.jpg]
