# Supplementary material for: Selection and validation of optimal reference genes for RT-qPCR analyses in Aphidoletes aphidimyza Rondani (Diptera: Cecidomyiidae)
Source: Front Physiol. 2023 Oct 25;14:1277942. doi: 10.3389/fphys.2023.1277942 (PMC10634233; doi:10.3389/fphys.2023.1277942)
Supplement: Supplementary file 2 [file Table1.docx]

**Table S1 Primers of eight candidate house-keeping genes for RT-PCR**

| Gene Name | Primer sequences (5' to 3´) | Amplicon size (bp) | Accession Number |
| --- | --- | --- | --- |
| $\beta$*-actin* | F-CGCTTTGGTTGTAGACAATG | 1081 | OP321086 |
|  | R-ACCGCTTTCGTCGTATTCTT |  |  |
| $\alpha$*-Tub* | F-GTAATGCCTGCTGGGAATTG | 1194 | OP321087 |
|  | R-CTTCCATACCTTCACCGACA |  |  |
| *EF1-*$\alpha$ | F-TGATCTACAAATGCGGTGGT | 1260 | OP321088 |
|  | R-GTGCATCCTTGAAGTTGACG |  |  |
| *RPL8* | F-TAGCACATACGAAAAAACGC | 669 | OP321090 |
|  | R-CTTTGTTTCTGCTTTACCAC |  |  |
| *RPL32* | F-ACCAGCTTACAGACCGAAAA | 387 | OP321091 |
|  | R-TTTTCTTGTGAACGAAGGCG |  |  |
| *RPS3* | F-ACGAAAGTTCGTCTTGAACG | 681 | OP321092 |
|  | R-CCAGCAATTTCTCGGATTGG |  |  |
| *RPS13* | F-GCTTTGCCATACCGTCGTTC | 374 | OP321093 |
|  | R-GCTGTGCTTGATTCGTATTT |  |  |
| *GAPDH* | F-ATGGATTTGGTCGCATTGGT | 936 | OP321089 |
|  | R-TAATAAGGTCGATAACACGG |  |  |
