# Supplementary material for: Selection and validation of optimal reference genes for RT-qPCR analyses in Aphidoletes aphidimyza Rondani (Diptera: Cecidomyiidae)
Source: Front Physiol. 2023 Oct 25;14:1277942. doi: 10.3389/fphys.2023.1277942 (PMC10634233; doi:10.3389/fphys.2023.1277942)
Supplement: Supplementary file 3 [file Table2.docx]

**Table S2 Primers of eight candidate house-keeping genes used in qRT-PCR**

| Gene Name | Primer sequences (5' to 3´) | Length (bp) | Slope | Regression coefficients | Mplification efficiency (%) |
| --- | --- | --- | --- | --- | --- |
| $\beta$*-actin* | F- GCCGTCTTTCCATCAATTGTC | 153 | -3.207 | 0.999 | 105.01 |
|  | R- CCAGTTGGTGATGATACCGT |  |  |  |  |
| $\alpha$*-Tub* | F-ACACCACATTGGAACACTCT | 171 | -3.424 | 0.997 | 95.90 |
|  | R-TAAGTGCTCCATCGAAACGA |  |  |  |  |
| *EF1-*$\alpha$ | F-TTCAAAGAACGGACAAACCC | 208 | -3.216 | 0.994 | 104.62 |
|  | R-CCATCCTGAGATTGGAACGA |  |  |  |  |
| *RPL8* | F-TGGAAAGAAAGCCACTTTGC | 236 | -3.310 | 0.997 | 100.50 |
|  | R-CAATACCAACCATAGCACGG |  |  |  |  |
| *RPL32* | F-GTCGTTTCAAGGGCCAATAC | 175 | -3.318 | 0.996 | 100.16 |
|  | R-GATGAAACGTTGTGGGCAAT |  |  |  |  |
| *RPS3* | F-ACGAAAGTTCGTCTTGAACG | 194 | -3.199 | 0.996 | 105.38 |
|  | R-CAACAGCTGTAAGTTCACGG |  |  |  |  |
| *RPS13* | F-CCAATCAGCTTTGCCATACC | 167 | -3.362 | 1.000 | 98.37 |
|  | R-CGCTAACAAAACGTACCTGG |  |  |  |  |
| *GAPDH* | F-GGTCCATCTGGAAAGCTATGG | 205 | -3.127 | 0.995 | 108.79 |
|  | R-TTTCGTCGTATTTGGCTGGT |  |  |  |  |
